# Eptifibatide use in ischemic stroke patients undergoing endovascular thrombectomy: A matched cohort analysis

**DOI:** 10.3389/fneur.2022.939215

**Published:** 2022-09-27

**Authors:** Ameena Rana, Siyuan Yu, Savina Reid-Herrera, Scott Kamen, Krystal Hunter, Hamza Shaikh, Tudor Jovin, Olga R. Thon, Parth Patel, James E. Siegler, Jesse M. Thon

**Affiliations:** ^1^Cooper Medical School of Rowan University, Camden, NJ, United States; ^2^Cooper Research Institute, Cooper University Hospital, Camden, NJ, United States; ^3^Department of Radiology, Cooper University Hospital, Camden, NJ, United States; ^4^Cooper Neurological Institute, Cooper University Hospital, Camden, NJ, United States

**Keywords:** stroke, thrombectomy, neurointervention, ischemic stroke, endovascular treatment

## Abstract

**Introduction:**

Small studies have suggested that eptifibatide (EPT) may be safe in acute ischemic stroke (AIS) following intravenous thrombolysis or during endovascular therapy (EVT) for large vessel occlusion (LVO). However, studies are called upon to better delineate the safety of EPT use during EVT.

**Methods:**

A comprehensive stroke center registry (09/2015-12/2020) of consecutive adults who had undergone EVT for anterior LVO was queried. Patients treated with EPT were matched with 2 control groups based on known factors associated with intracranial hemorrhage (ICH) risk - age, Alberta Stroke Program Early Computed Tomography Score (ASPECTS), and number of thrombectomy passes. Safety outcomes (intracranial hemorrhage [ICH], parenchymal hematoma [PH-2] grade hemorrhagic transformation, symptomatic ICH [sICH]) and efficacy outcomes (TICI 2B/3 recanalization, 24-h National Institutes of Health Stroke Scale [NIHSS] score), were compared between matched groups using descriptive statistics. In addition, multivariable logistic regression was used to assess for an association between EPT and PH-1/PH-2 grade hemorrhages.

**Results:**

A total of 162 patients were included, 54 of whom (33%) received EPT. The rate of ICH was similar between groups (*p* = 0.62), while PH-2 was significantly more frequent with EPT (16.7% EPT vs. 3.7 vs. 1.9%; *p* = 0.009), but without significant differences in sICH (5.6% EPT vs. 7.4 vs. 3.7%; *p* = 0.72). Rates of TICI Score ≥ 2B were nominally higher with EPT use (83.3 vs. 77.8 vs. 77.8%, *p* = 0.70). Between the EPT and control groups, there were no differences in 24-h NIHSS (*p* = 0.09) or 90-day mortality (*p* = 0.58). Our adjusted multivariate analysis identified that the number of passes (*p* < 0.01), EPT use (*p* < 0.01), and tandem occlusion (*p* = 0.03) were independent predictors of PH1/PH2 grade hemorrhage. Additionally, every unit increase in number of passes resulted in a 1.5 times greater odds of a high-grade hemorrhagic transformation in EPT-treated patients (adjusted OR = 1.594).

**Conclusion:**

In this single-center analysis, EPT use during EVT was associated with a significantly higher rate of PH1/PH2 grade hemorrhages, but not with differences in sICH, 24-h NIHSS, or 90-day mortality. Randomized prospective trials are needed to determine the safety and efficacy of EPT in this population.

## Introduction

Stroke is the eighth most common cause of death worldwide and the number one cause of disability within the United States (1). Endovascular therapy (EVT) is the standard of treatment for ischemic stroke due to large vessel occlusion (2), however in 2–20% of cases, patients experience reocclusion of the treated vessel after initial recanalization. Predictors of reocclusion include site of occlusion, more complex procedure such as those requiring multiple passes, atherosclerotic etiology of stroke, or residual thrombus or stenosis after recanalization attempt (3). In this subset of patients with high risk of reocclusion, or in cases of difficulty with initial recanalization, emergent antithrombotic therapies, such as eptifibatide (EPT), are often utilized (3).

EPT is a glycoprotein IIB/IIIA receptor antagonist that was first used in combination with reduced-dose fibrinolytic agent for treatment of myocardial infarction (4). Since then, multiple studies have tested the safety and efficacy of EPT in various combinations with recombinant tissue plasminogen activator (tPA). CLEAR, a 2008 multicenter randomized controlled trial, was one of the first studies to demonstrate the safety of EPT use in combination with low-dose recombinant tissue plasminogen activator (tPA). The study found no increased risk of symptomatic intracranial hemorrhage (sICH) when compared with full-dose tPA for the treatment of ischemic stroke (5). Subsequently, both the CLEAR-ER and CLEAR-FDR also demonstrated the comparable safety of EPT with full-dose tPA (6, 7).

More recently, the use of EPT in EVT for carotid occlusions with stent placement and for tandem occlusions has been investigated (3, 8). However, these studies did not include matched control groups of patients who did not receive EPT, and thus safety comparisons could not be made. The objective of our study is to examine the safety and efficacy of EPT when used during EVT using a matched control analysis.

## Methods

Data will be made available to any qualified investigator upon reasonable request.

### Study design and data acquisition

A Comprehensive Stroke Center registry (09/2015–12/2020) of consecutive adults who had undergone endovascular thrombectomy for large vessel occlusion was queried for this nested cohort study. Patient data was abstracted retrospectively between 09/2015 and 08/2019, and prospectively from 09/2019–12/2020 as part of a larger, prospective observational registry. Patients treated for acute anterior large vessel occlusion (LVO affecting the internal carotid artery, M1, or M2 branches of the middle cerebral artery) were screened for inclusion in a matched analysis. Two control groups were necessary to maintain equivalence in patient sample size across each group (*n* = 54) and maintain a match of 1:2 for our matched cohort study design. Given that our study outcome (ICH Grade PH1/2) has a low rate of incidence (13.5% in our sample), it was necessary an additional control group be added in order to increase the sample and stabilize the data. There were a total of 291 who were within our inclusion criteria, of which, 54 were given EPT. We then selected controls by matching the remaining by age (within 10 years), Alberta Stroke Program Early Computed Tomography Score (ASPECTS, less than or greater than 6), and number of thrombectomy passes (dichotomized by 1 vs. >1) to the EPT cases. By this random process, the first two controls which were shown to match with each respective EPT case were included in the study sample.

### Variables and statistical analysis

Demographic data including patient's age, sex, race and ethnicity, pre-morbid modified Rankin Score (mRS), baseline National Institutes of Health Stroke Scale (NIHSS) score, systolic blood pressure on arrival, and hematologic parameters were collected for all patients. The indication for eptifibatide use was made at the discretion of the neurointerventionist, and included difficulty with or incomplete recanalization, re-occlusion, and/or distal embolization, although the indication for each patient was not available. Eptifibatide was administered as a 180 ug/kg intravenous bolus. Procedural variables were abstracted from the electronic medical record or adjudicated using independent verification of neuroimaging by a board-certified interventional neuroradiologist or vascular neurologist, and included site of arterial occlusion, concurrent treatment with intravenous thrombolysis, number of thrombectomy passes, and final thrombolysis in cerebral infarction (TICI) score.

Safety outcomes were adjudicated by manual review of neuroimaging by a vascular neurologist who was blinded to the treatment and included the presence of any intracerebral hemorrhage (ICH) on follow-up imaging, symptomatic intracerebral hemorrhage [sICH, by ECASS III criteria (9), defined as deterioration in NIHSS score by ≥4 points that was attributed to the intracranial hemorrhage]. Hemorrhagic transformation (HT) grade was defined and classified into four subtypes: hemorrhagic infarct Type 1 (HI-1), hemorrhagic infarct Type 2 (HI-2), parenchymal hematoma Type 1 (PH-1), and parenchymal hematoma Type 2 (PH-2) as previously described (10). MRI was used to grade HT except in cases where clinical symptoms necessitated emergent neuroimaging or when MRI could not be performed, in which cases head CT was used. All sICH events were adjudicated by a second vascular neurologist using a modified Delphi consensus. Efficacy outcomes included final TICI score, with a score of 2B, 2C or 3 considered successful recanalization, 24-h NIHSS score, and 90-day mortality. Discharge disposition (home, acute inpatient rehabilitation facility, skilled nursing facility/long-term acute care [SNF/LTAC], hospice, and in-hospital death) were also evaluated. Hospice care refers to inpatient or home care intended to provide comfort and quality of life when the prognosis for a meaningful recovery is low.

Patients were matched based on age, number of passes and ASPECTS. The patient's age was converted into an ordinal scale. Pass numbers were dichotomized into one or more than one pass and ASPECTS were dichotomized into 0–5 group and 6–10 group. After creating these variables, patients in the EPT group with similar characteristics were matched manually in a 1:2 fashion with patients in the control group. Matched patient groups were compared using Friedman's test and Cochran's Q.

To comprehensively evaluate the relationship between EPT and PH1/PH2 as a secondary outcome, we generated 4 adjusted logistic regression models. In the first model (Model A), all clinical and radiographic variables from Table 1 were included in the adjusted regression. The second model (Model B) was adjusted based on strength and significance of association from univariate regression (*p* < 0.05). The third model (Model C) was run with explanatory variables that were shown to be important according to a gradient boosting model (GBM). GBM is a data classifier that uses decision tree analysis to score data elements in order to determine the data elements that make the greatest contribution for respective models. The final model (Model D) run was based on biological plausibility, including pre-specified variables that are predictive of PH1/PH2. Effect estimates are summarized using odds ratios (OR) with corresponding 95% confidence intervals (CI).

**Table 1:** Baseline patient characteristics.

	**Eptifibatide**	**First control**	**Second control**	***P*-value**
	**(*n =* 54)**	**(*n =* 54)**	**(*n =* 54)**	
Age, median y (IQR)	67 (59–73)	68 (60–77)	68 (59–77)	0.55
Female, no. (%)	35 (64.8%)	30 (55.6%)	26 (48.1%)	0.21
Race, no. (%)				
Caucasian	30 (55.6%)	30 (55.6%)	40 (74.1%)	0.07
African American	13 (24.1%)	14 (25.9%)	10 (18.5%)	0.63
Asian/Pacific Islander	2 (3.7%)	2 (3.7%)	0 (0%)	0.37
Other	4 (7.4%)	2 (3.7%)	0 (0%)	0.14
Hispanic, no. (%)	3 (5.6%)	6 (12.5%)	4 (7.4%)	0.42
ASPECTS, median (IQR)	9 (8–10)	9 (9–10)	9 (8–10)	0.08
Pre-morbid mRS score, median (IQR) (*n =* 42^*^)	0 (0–1)	0 (0–1)	0 (0–0)	0.29
Pre-morbid mRS 0–2				
no. (%)	40/42 (95.2%)	36/42 (85.7%)	39/42 (92.9%)	0.24
Baseline NIHSS, median (IQR)	13 (9–19)	16 (9–21)	14 (9–20)	0.61
Wake-up stroke no. (%)	15 (27.8%)	20 (37.0%)	8 (14.8%)	0.03
Last known well to arrival, median min (IQR) (*N =* 44^*^)	461 (177–728)	305 (132–596)	281 (180–454)	0.43
Systolic blood pressure on arrival, median mmHg (IQR) (*N =* 51^*^)	161 (130–189)	156 (130–186)	141 (125–165)	0.14
Platelet count, median (IQR) (*N =* 52^*^)	236 (188–269)	216 (181–279)	220 (175–296)	0.39
International normalized ratio, median (IQR) (*N =* 41^*^)	1.1 (1–1.2)	1.2 (1.1–1.4)	1.1 (1–1.2)	0.39
Hematocrit, median (IQR) (*N =* 47^*^)	38.6 (36–41)	38.5 (33.4–42.4)	38.6 (36.3–43)	0.76
Site of occlusion, no. (%)				
Internal carotid artery	29 (53.7%)	19 (35.2%)	14 (25.9%)	0.010
Middle cerebral artery	36 (66.7%)	39 (72.2%)	40 (74.1%)	0.68
Tandem	14 (25.9%)	8 (14.8%)	5 (9.3%)	0.065
Past Medical History, no. (%)				
Hypertension	28 (51.9%)	38 (70.4%)	43 (79.6%)	0.009
Hyperlipidemia	15 (27.8%)	23 (42.6%)	25 (46.3.%)	0.13
Diabetes	15 (27.8%)	15 (27.8%)	17 (31.5%)	0.89
Prior Stroke/Transient ischemic attack	12 (22.2%)	6 (11.1%)	11 (20.4%)	0.30
Atrial fibrillation	4 (7.4%)	17 (31.5%)	15 (27.8%)	0.0003
Peripheral vascular disease	4 (7.4%)	3 (5.6%)	3 (5.6%)	0.89
Tobacco use	23 (42.6 %)	19 (35.2%)	14 (25.9%)	0.18
Prior antiplatelet and anticoagulation use				
Prior antiplatelet use, no. (%)	12 (22.2%)	20 (37.0%)	19 (35.2%)	0.23
Prior aspirin use, no. (%)	11 (20.4%)	19 (35.2%)	18 (33.3%)	0.21
Prior anticoagulation use, no. (%)	5 (9.3%)	12 (22.2%)	7 (13.0%)	0.13
Thrombolysis administration	14 (25.9%)	20 (37.0%)	16 (29.6%)	0.44
Suspected stroke etiology, no. (%)				
Large artery atherosclerosis	19 (47.5%)	5(12.5%)	5 (12.5%)	0.001
Cardioembolism	3 (7.5%)	19(47.5%)	16 (40.0%)	0.001
Other determined etiology/ undetermined etiology	18 (42.9%)	17 (40.5%)	19 (45.2%)	0.89

* Number of patients in each study group. IQR: interquartile range, ASPECTS Alberta stroke program early CT score; mRS modified Rankin scale, and NIHSS National Institutes of Health Stroke Scale.

All tests were performed at the two-sided level with an alpha set at 0.05. Missing data were not imputed. No adjustments were made for multiple comparisons. This investigation was approved by the local institutional review board with waiver of informed consent. These results are reported in accordance with Strengthening the Reporting of Observational Studies in Epidemiology guidelines. Analyses were performed using R and SPSS.

## Results

A total of 365 patients were treated with endovascular thrombectomy during the study period, of whom 291 patients had adjudicated ASPECTS. After matching for age, number of passes (1 or >1) and ASPECTS, 54 EPT patients were matched with 108 controls split between 2 control groups. The baseline characteristics of the patients are shown in Table 1. There were no significant differences between patients in the EPT and two control cohorts with regard to age, sex, race, median ASPECTS, or baseline mRS score. Baseline NIHSS score upon hospital presentation was nominally higher in the two control groups when compared to the EPT group (13 EPT vs. 15.5 vs. 14.5; *p* = 0.612). There were more ICA occlusions in the EPT group (53.7 vs. 35.2 vs. 25.9%; *p* = 0.010). Patients in the EPT group had significantly lower rates of hypertension (51.9 vs. 70.4 vs. 79.6%; *p* = 0.009) and atrial fibrillation (7.4 vs. 31.5 vs. 27.8%; *p* = 0.003). There were no significant differences between groups with regard to prior aspirin, antiplatelet, or anticoagulation use at the time of presentation. Additionally, there was no significant difference in the last known well time to hospital arrival, systolic blood pressure on arrival, baseline coagulopathy, or hematocrit level.

### Thrombectomy parameters

Large artery atherosclerosis was the most common etiology of stroke in patients receiving EPT (47.5 vs. 12.5 vs. 12.5%; *p* < 0.001), whereas cardioembolism was the most common etiology of stroke for the control groups (7.5 vs. 47.5 vs. 40%; *p* < 0.001). There was no difference in the number of passes between the EPT and control groups (2 vs. 2 vs. 2; *p* = 0.792). As outlined in Table 2, no statistically significant differences were seen in the number of patients achieving TICI score equal to or greater than 2B between the EPT and control groups, although rates were nominally higher in the EPT group (83.3 vs. 77.8 vs. 77.8%, *p* = 0.70). On follow-up imaging, there was no difference in rate of ICH among the groups. When divided by ICH grade, EPT patients had significantly lower rates of HI-1 ICH (9.3 vs. 29.6 vs. 16.7%; *p* = 0.028) but significantly higher PH-2 ICH grade when compared to the control groups (16.7 vs. 3.7 vs. 1.9%; *p* = 0.009).

**Table 2:** Endovascular treatment outcomes and grades of hemorrhagic transformation on subsequent imaging.

	**Eptifibatide**	**First control**	**Second control**	***P*-value**
	**(*n =* 54)**	**(*n =* 54)**	**(*n =* 54)**	
Number of passes, median (IQR)	2 (1–2)	2 (1–3)	2 (1–3)	0.79
TICI Score ≥ 2B, no. (%)	45 (83.3%)	42 (77.8%)	42 (77.8%)	0.70
ICH on follow up imaging, no. (%)	26 (48.1%)	27 (50.0%)	22 (40.7%)	0.62
ICH grade, no. (%)				
HI-1	5 (9.3%)	16 (29.6%)	9 (16.7%)	0.028
HI-2	6 (11.1%)	4 (7.4%)	4 (7.4%)	0.72
PH-1	6 (11.1%)	3 (5.6%)	1 (1.9%)	0.15
PH-2	9 (16.7%)	2 (3.7%)	1 (1.9%)	0.009
Symptomatic ICH, no. (%)	3 (5.6%)	4 (7.4%)	2(3.7%)	0.72

IQR, interquartile range; TICI, thrombolysis in cerebral infarction; ICH, intracerebral hemorrhage; HI, hemorrhagic infarction; PH, parenchymal hematoma.

For the primary outcome of symptomatic ICH, there was no significant difference among the groups (EPT 5.6 vs. 7.4 vs 3.7%; *p* = 0.717). As shown in Table 3, the median 24-h NIHSS score was 10 in the EPT group vs. 6 vs. 4 in the control groups, *p* = 0.09. No significant difference was seen in patients discharged home across groups. More patients in the second control group were discharged to rehab compared to EPT and first control (50 EPT vs. 25.9 vs. 64.8%; *p* = 0.001). More patients in the second control cohort and EPT group were discharged to SNF or LTAC for further treatment (9.3 vs. 0 vs. 13%; *p* = 0.039). The first control cohort also had significantly higher discharge to hospice (3.7 vs. 20.4 vs. 3.7%; *p* = 0.003) and in-hospital death (13 vs. 16.7 vs. 0%; *p* = 0.011). Additionally, no difference in 90-day mortality was seen (15.1 vs. 22.6 vs. 20.8%, *p* = 0.58).

**Table 3:** Discharge and 90 day outcomes.

	**Eptifibatide**	**First control**	**Second control**	***P-*value**
24 h NIHSS, median (IQR) (*N =* 54^*^)	10 (4–18)	6 (5–16)	4 (2–11)	0.09
Discharge, no. (%) (*N =* 54^*^)				
Home	13 (24.1%)	20 (37.0%)	10 (18.5%)	0.09
Acute rehab	27 (50.0%)	14 (25.9%)	35 (64.8%)	< 0.01
SNF/ LTAC	5 (9.3%)	0 (0.0%)	7 (13%)	0.04
Hospice	2 (3.7%)	11 (20.4%)	2 (3.7%)	< 0.01
Death	7 (13%)	11 (20.4%)	0 (0%)	0.01
Mortality at 90 days, no. (%) (*N =* 53^*^)	8/53 (15.1%)	12/53 (22.6%)	11/53 (20.8%)	0.58

* Number of patients in each study group.

NIHSS, National Institutes of Health Stroke Scale; IQR, interquartile range; SNF, skilled nursing facility; LTAC, long-term acute care.

### Regression analysis

Univariate models and multiple multivariate models were generated to identify the relationship between age, ASPECTS, number of passes, EPT use, tandem occlusions, intravenous thrombolysis (IVT) administration and risk of post-operative PH1 and PH2 grade hemorrhagic transformation (Table 4). In univariate analysis, age, ASPECTS, tandem occlusion, and IVT administration were not associated with PH1/PH2 grading on post-operative imaging. Number of passes (OR: 1.525; 95% CI 1.049–2.216; *p* = 0.027) and EPT use (OR: 5.549; 95% CI 2.103–14.643; *p* < 0.001) were associated with PH1/PH2 grade hemorrhages.

**Table 4:** Univariate and multivariable models for independent predictors of PH-1 and PH-2 after mechanical thrombectomy.

	**Unadjusted** ^*^	**Model A** ^**^	**Model B** ^***^	**Model C** ^***^	**Model D** ^****^
Eptifibatide use	OR: 5.549 95% CI 2.103–14.643 *p < * 0.001	OR: 8.947 95% CI 1.565–51.143 *P =* 0.014	OR: 10.54 95% CI 3.266–34.011 *P =* 0.0001	OR: 5.683 95% CI 1.809–17.850 *P =* 0.003	OR: 6.130 95% CI 2.239–16.782 *P =* 0.0001
Age	OR: 0.999 95% CI 0.961–1.039 *p =* 0.961	OR: 0.964 95% CI 0.893–1.042 *p =* 0.358	Nonsignificant factor: based on Model A	OR: 0.995 95% CI 0.946–1.045 *p =* 0.995	0.921 95% CI 0.605–1.401 *p =* 0.921
ASPECTS	OR: 1.025 95% CI 0.681–1.544 *p =* 0.904	OR: 0.748 95% CI 0.403–1.388 *p =* 0.358	Nonsignificant factor: based on Model A	OR: 1.083 95% CI 0.653–1.794 *p =* 0.079	OR: 1.160 95% CI 0.743–1.810 *p =* 0.513
Pass number	OR: 1.525 95% CI 1.049–2.216 *P =* 0.027	OR: 2.665 95% CI 1.304–5.447 *p =* 0.007	OR: 1.826 95% CI 1.183–2.816 *P =* 0.006	OR: 1.738 95% CI 1.074–2.810 *p =* 0.024	OR: 1.594 95% CI 1.064–2.388 *p =* 0.024
Tandem occlusion	OR: 0.209 95% 0.027–1.623 *P =* 0.134	OR: 0.031 95%CI 0.002–0.621 *p =* 0.023	OR: 0.078 95% CI 0.008–0.728 *P =* 0.025		
Thrombolysis administration	OR: 2.083 95% CI 0.834–5.206 *P =* 0.116	OR: 7.404 95% CI 1.166–47.033 *P =* 0.034	OR: 2.683 95% CI 0.890–8.090 *P =* 0.080		
Initial systolic blood pressure	OR: 0.991 95% CI 0.978–1.005 *P =* 0.231	OR: 0.957 95% CI 0.926–0.988 *P =* 0.007	OR: 0.983 95% CI 0.966–1.000 *P =* 0.0054	OR: 1.002 95% CI 0.905–1.010 *P =* 0.497	

* Unadjusted model: Age, Gender (female), Race (Caucasian), baseline NIHSS, systolic blood pressure, hematocrit, INR, ICA occlusion, MCA occlusion, tandem occlusion, coronary artery disease, atrial fibrillation, smoking, hypertension, diabetes mellitus, peripheral vascular disease, prior stroke, any antiplatelet use, atherosclerosis.

** Model A: Adjusted analysis: Female sex, Race (Caucasian), Baseline NIHSS, Systolic blood pressure, hematocrit, INR, ICA occlusion, tandem occlusion, coronary artery disease, heart failure, atrial fibrillation, smoking, hypertension, diabetes mellitus, peripheral vascular disease, prior stroke, antiplatelet use prior to hospitalization, atherosclerosis.

*** Model B: Adjusted model based on significant findings in model A (*p* < 0.05 in univariate regression).

^***^Model C: Adjusted based on logistic regression tree analysis: platelet count, systolic blood pressure at admission, baseline hematocrit, baseline NIHSS, INR, coronary artery disease, ICA occlusion, atherosclerosis, female sex, hyperlipidemia, diabetes.

**** Model D: Adjusted based on pre-specified exposure variables which have the most biologic plausibility for predicting the outcome of parenchymal hematoma grades I or II, which included age, ASPECTS score, number of passes and eptifibatide use.

OR: odds ratio, CI confidence interval, ASPECTS Alberta Stroke Program Early Computed Tomography Scale, NIHSS National Institutes of Health Stroke Scale, INR international normalized ratio, ICA internal carotid artery, and MCA middle cerebral artery.

Three adjusted multivariate models were used to assess risk factors for PH1/PH2 grade hemorrhages post thrombectomy. Model A used an adjusted multivariate analysis which showed that number of passes (OR: 2.665; 95% CI 1.304–5.447; *p* = 0.007), EPT use (OR: 8.947; 95% CI 1.565–51.143; *p* = 0.014), tandem occlusions (OR: 0.031; 95% CI 0.002–0.621; *p* = 0.023), IVT administration (OR: 7.404; 95% CI 1.166–47.033; *p* = 0.034) and initial systolic blood pressure (OR: 0.957; 95% CI 0.926–0.988; *p* = 0.007) were associated with PH1/PH2 grade hemorrhage.

Model B utilized significant variables based on model A. Number of passes (OR: 1.826; 95% CI 1.183–2.816, *p* = 0.006), EPT (OR: 10.54; 95% CI 3.266–34.011, *p* = 0.0001) and tandem occlusion (OR: 0.078; 95% CI 0.008–0.728, *p* = 0.025) remained independent predictors of PH1/PH2 grade hemorrhage.

Model C used a logistic regression tree analysis which showed that number of passes (OR: 1.738; 95% CI 1.074–2.810, *p* = 0.024) and EPT (OR: 5.683; 95% CI 1.809–17.850, *p* = 0.003) were associated with PH1/PH2 grade hemorrhage. In Model D, variables were added to the logistic regression model based on biological plausibility and association with hemorrhagic transformation, including age, ASPECTs, number of passes, and EPT. In this model, number of passes (OR: 1.594; 95% CI 1.064–2.388, *p* = 0.024) and EPT (OR: 6.130; 95% CI 2.239–16.782, *p* = 0.0001) were independently associated with PH1/PH2 hemorrhage.

## Discussion

In difficult cases of mechanical thrombectomy or to prevent re-occlusion following successful large artery recanalization in acute ischemic stroke, GP IIB/IIIA receptor antagonists have been utilized, the most common agent being EPT. However, there is a theoretical increased risk of intracranial hemorrhage associated with the use of this antiplatelet agent, which has been incompletely evaluated to date. Given the recent and emerging data showing the efficacy of mechanical thrombectomy in a growing population of patients with LVO-associated stroke (11), it is increasingly important to delineate the safety and efficacy profiles of treatments used for EVT (12).

Recent small retrospective studies evaluating the use of EPT in EVT have shown encouraging results regarding the safety of EPT in this setting. Osteraas et al. described 29 patients that were treated with EPT during carotid EVT and stent placement, 21 of whom had an intracranial tandem occlusion. They found low rates of sICH, including in those who were treated with IV tPA prior to EVT (3). Jost et al. described 58 patients who were treated with EPT during EVT of tandem large artery occlusions and similarly showed low rates of sICH in this population (8). However, both of these studies were descriptive and only included EPT-treated patients without matched controls.

Our study evaluated the safety of EPT use in patients undergoing EVT compared to control groups that did not receive EPT and were matched based on factors previously shown to be associated with increased intracranial hemorrhage risk - age, ASPECTS, and number of thrombectomy passes (13–15). We found that EPT was not significantly associated with higher rates of sICH, as defined by European Cooperative Acute Stroke Study (ECASS)-II criteria (16). Our study also showed no statistically significant differences in 24-h NIHSS score or 90-day mortality between the EPT group vs. control groups that received EVT alone.

The EPT-treated patients in our study had a significantly higher rate of PH2-grade hemorrhagic transformation, which prior studies have shown to be correlated with early deterioration and 3-month mortality (17–19). In contrast to these studies, we did not find a significant difference in 3-month outcomes in those with PH2 hemorrhages. Further adjusted analysis of the data using a multivariate model for independent predictors of PH1 and PH2 hemorrhage after mechanical thrombectomy showed a statistically significant correlation between EPT use, number of passes during thrombectomy, IVT administration, and tandem occlusion stroke in the occurrence of these hemorrhages, as shown in Table 4. Our multivariate analysis showed that every unit increase in passes resulted in a 1.5 times greater odds of a high-grade ICH in EPT-treated patients (*p* = 0.03).

The greatest strength of our study is the sample size of 162 patients, the largest in this population to date, and its matched cohort design utilizing risk factors for ICH.

Limitations of our study that could potentially introduce biases include its retrospective nature, patient population from a single-center, and heterogeneity of individual EPT treatment indications. Additionally, EPT was administered as an IV bolus, and our results may not be applicable to other dosing regimens. Aside from 90-day mortality data, other functional outcome metrics were available in a minority of patients and thus not compared. Although the groups were overall well-matched, there were a few differences between baseline patient characteristics. The EPT group had a greater percentage of large artery atherosclerosis as the stroke etiology and the control groups had higher rates of cardioembolism as the stroke mechanism of LVO. However, these factors have not been shown to be associated with increased ICH risk following EVT, making their relevance less likely (14, 15). While these factors do not have an increased ICH risk following EVT, the difference in the stroke mechanism between the EPT and control groups may have contributed to the selection of treatment in these patients which is another limitation of our study. While the use of acute intracranial stenting is infrequently performed in our institution, we recently showed the safety and potential efficacy of acute rescue stenting in large vessel occlusion in a multi-center observational analysis (20). There was also heterogeneity between the groups (including between the control groups) regarding discharge disposition, precluding interpretation of these results.

Larger, prospective studies are needed to support the findings from this study and to better define the optimal dosing of EPT during EVT, as well as to further delineate the safety of EPT use with other antithrombotic agents and acute treatments for ischemic stroke.

## Conclusions

In this single-center matched cohort analysis, EPT use during EVT was associated with a higher rate of PH2 grade hemorrhagic transformation but not with differences in sICH, 24-hour NIHSS score, or 90-day mortality. These data suggest that EPT may be safe during EVT and support the utility of a larger randomized clinical trial, or pooling of observational cohort data, to evaluate the safety and efficacy of EPT use during EVT.

## Data availability statement

The raw data supporting the conclusions of this article will be made available by the authors, without undue reservation.

## Author contributions

AR and SY collected data, analyzed data, and drafted manuscript. SR-H, SK, and HS collected and analyzed data. KH statistical analysis of data. TJ adjudicated data and edited manuscript. OT analyzed data and edited manuscript. PP collected data. JS and JT collected, analyzed, adjudicated data, and edited manuscript. All authors contributed to the article and approved the submitted version.

## Conflict of interest

The authors declare that the research was conducted in the absence of any commercial or financial relationships that could be construed as a potential conflict of interest.

## Publisher's note

All claims expressed in this article are solely those of the authors and do not necessarily represent those of their affiliated organizations, or those of the publisher, the editors and the reviewers. Any product that may be evaluated in this article, or claim that may be made by its manufacturer, is not guaranteed or endorsed by the publisher.
